# Response to Trastuzumab Deruxtecan in a Patient With *EGFR*-Mutated NSCLC and Transformation to SCLC Harboring *HER2* Amplification: A Case Report

**DOI:** 10.1016/j.jtocrr.2026.101013

**Published:** 2026-05-02

**Authors:** Malte Verheyen, Lea Ruge, Anne Bunck, Felix John, Lucia Nogova, Richard Riedel, Sofia Stilianakis, Heather Scharpenseel, Matthias Scheffler, Idil Ahmed, Carolin Jakob, Alexander Quaas, Jan Philip Weber, Martin Sebastian, Fabian Acker, Jürgen Wolf, Sebastian Michels

**Affiliations:** a University of Cologne, Faculty of Medicine and University Hospital Cologne, Department I of Internal Medicine, Center for Integrated Oncology Aachen Bonn Cologne Duesseldorf, Lung Cancer Group Cologne (LCGC), Cologne, Germany; b Department of Radiology, University of Cologne, Cologne, Germany; c Institute of Pathology, University Hospital Cologne, Cologne, Germany; d MV-Center for Oncology and Hematology, Sachsenring, Cologne, Germany; e Department of Medicine II, Hematology and Oncology, Frankfurt Cancer Institute, University Hospital Frankfurt, Frankfurt, Germany

**Keywords:** EGFR-mutant NSCLC, Acquired resistance, Small cell transformation (SCT), HER2 amplification, Trastuzumab deruxtecan, Case report

## Abstract

Small cell transformation (SCT) represents a rare mechanism of acquired resistance to EGFR inhibitors in *EGFR*-mutant NSCLC and is associated with poor prognosis. *HER2* amplification constitutes another less frequent resistance mechanism, acting as a bypass of EGFR signaling. The coexistence of SCT and *HER2* amplification has not been previously described and is exceedingly rare. We report a patient with *EGFR*-mutant NSCLC who developed concurrent SCT and *HER2* amplification during treatment with osimertinib. Following rapid disease progression on chemoimmunotherapy, the patient achieved clinically meaningful and radiographic response (Response Evaluation Criteria in Solid Tumors version 1.1) to trastuzumab deruxtecan.

## Introduction

Third-generation EGFR tyrosine kinase inhibitors (TKIs) have significantly improved outcomes in *EGFR*-mutant NSCLC, but acquired resistance is inevitable. Histologic transformation to SCLC is a well-recognized acquired resistance mechanism, occurring in a rare yet clinically relevant subset of patients. Lacking effective evidence-based therapeutic approaches, it is associated with poor prognosis and is typically managed using platinum-based chemotherapy. *HER2* amplification represents another mechanism of resistance, although less frequently observed, further limiting the efficacy of EGFR-directed therapies. The coexistence of small cell transformation (SCT) and *HER2* amplification in a single patient is exceptionally rare. Although trastuzumab deruxtecan (T-DXd) has demonstrated efficacy in *HER2*-driven NSCLC, its role in transformed SCLC remains undefined. Here, we report a case of *EGFR*-mutant NSCLC with SCT and concomitant *HER2* amplification that responded to T-DXd.

## Case Presentation

**Figure 1:**
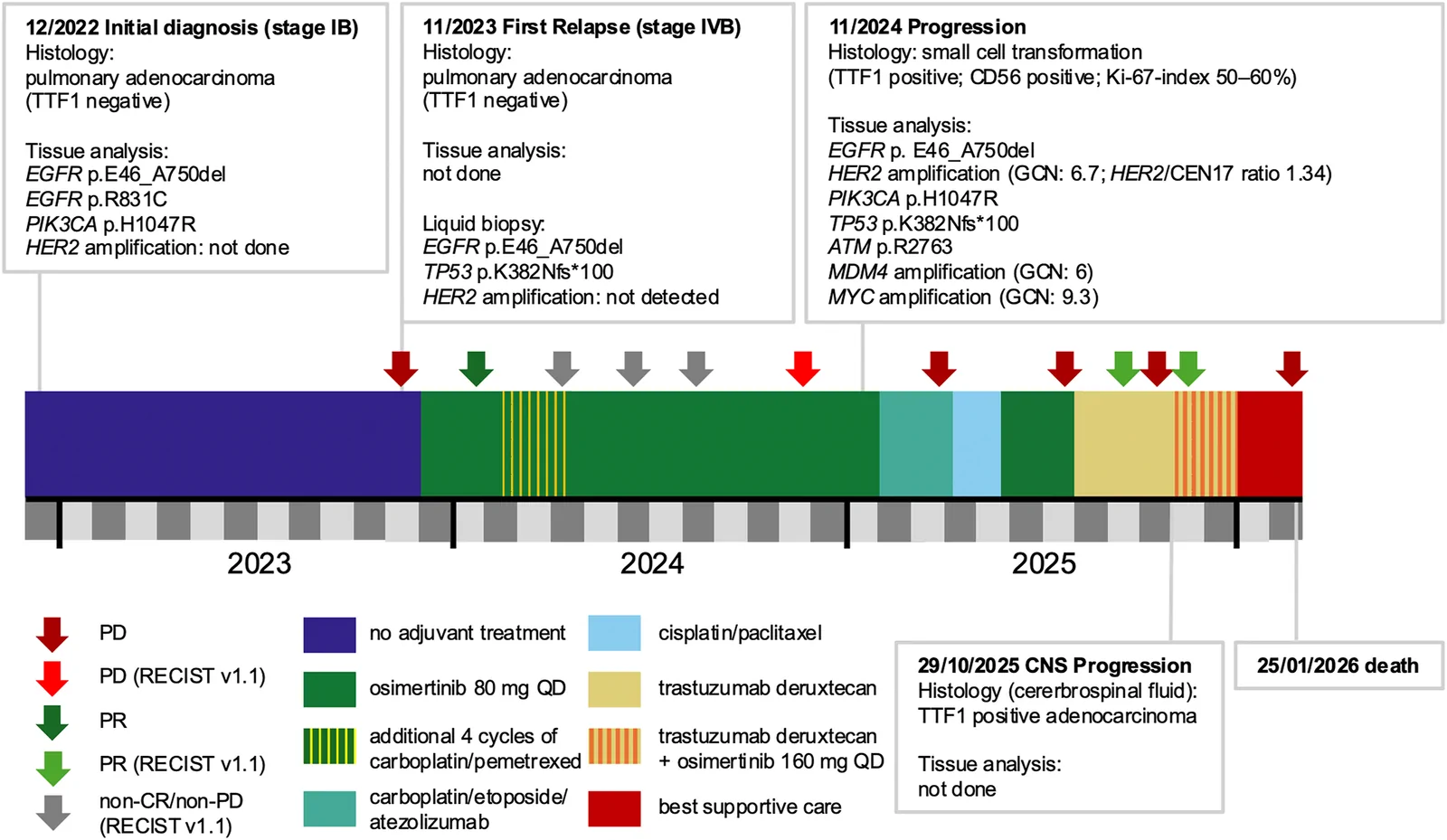
Treatment overview. Timeline of systemic therapies with rebiopsies and corresponding histopathologic and molecular alterations. Each bar represents 1 month, and response to therapy is marked with arrows that are color coded according to response and response evaluation (dark red, PD assessed by local radiologist according to standard of care; bright red, PD assessed by local radiologist according to RECIST v.1.1; dark green, PR assessed by local radiologist according to standard of care; bright green, PR assessed by local radiologist according to RECIST v.1.1; gray, non-CR/non-PD assessed by local radiologist according to RECIST v.1.1). CR, complete response; FISH, fluorescence in situ hybridization; GCN, gene copy number; PD, progressive disease; PR, partial response; RECIST v.1.1, Response Evaluation Criteria in Solid Tumors version 1.

A 63-year-old never-smoking woman was diagnosed with having Union for International Cancer Control stage IB (eighth edition) pulmonary adenocarcinoma in December 2022 (Fig. 1) and underwent right lower lobectomy. Histopathologic assessment confirmed a moderately differentiated, TTF1-negative adenocarcinoma. Molecular profiling was performed using multiplex polymerase chain reaction–based, amplicon next-generation sequencing (NGS) with a customized gene panel. Libraries were prepared using the QIAseq Targeted DNA Panel version (v.) 3 (QIAGEN, Hilden, Germany), and sequencing was performed on an Illumina MiSeq platform. This analysis revealed an *EGFR* exon 19 deletion (p.E746_A750del). Following R0 resection, no adjuvant EGFR-targeted therapy was initiated, as it was not guideline recommended at the time.

In November 2023, the patient presented with a right-sided pleural effusion, and cytologic assessment confirmed pleural carcinomatosis of the previously diagnosed TTF1-negative adenocarcinoma. Molecular analysis was not repeated, as the *EGFR* mutation was already known. Further staging revealed osseous metastases, classifying the disease as stage IVB. Osimertinib 80 mg once daily was initiated. The first restaging computed tomography (CT) at the end of November 2024 demonstrated regression of hilar and axillary lymph nodes and decreased pleural effusion.

The patient was prescreened for the phase II PACE-LUNG trial (NCT05281406), which evaluates the addition of chemotherapy in patients with persistent plasma circulating tumor DNA (ctDNA) *EGFR* mutations after initiation of first-line osimertinib. ctDNA analysis was performed using hybrid-capture NGS (Guardant360 CDx assay; Guardant Health, Redwood City, CA) before osimertinib therapy and at week 3, demonstrating persistent *EGFR* mutation without detection of *HER2* amplification. The patient subsequently received four cycles of carboplatin and pemetrexed in combination with osimertinib. Best objective response was stable disease (Response Evaluation Criteria in Solid Tumors [RECIST] v.1.1: non–complete response/non–progressive disease of nontarget lesions; no assessable target lesions). ctDNA analysis in June 2024, after completion of chemotherapy, revealed no detectable *EGFR* mutation or other molecular alterations. Osimertinib monotherapy was continued per study protocol.

In November 2024, CT scans revealed new pulmonary lesions consistent with progressive disease (RECIST v.1.1). Biopsy result of a right upper lobe metastasis revealed SCLC with a Ki-67 index of 50% to 60%. Comprehensive molecular profiling using hybrid-capture NGS (TruSight Oncology 500 panel, Illumina, San Diego, CA) revealed persistence of the known *EGFR* exon 19 deletion, consistent with SCT as an acquired resistance mechanism to osimertinib. In addition, a newly detected TTF1 positivity was observed, but RB1 expression was retained. *HER2* amplification was not detected in the NGS assay. Given the higher sensitivity of fluorescence in situ hybridization for copy number assessment, *HER2* status was also evaluated by fluorescence in situ hybridization, which revealed a co-occurring *HER2* amplification (gene copy number: 6.7, *HER2*/CEN17 ratio: 1.34). Retrospective immunohistochemical analysis did not reveal HER2 overexpression (U.S. Food and Drug Administration–approved ready-to-use antibody [anti-Her2/neu rabbit monoclonal primary antibody, clone 4B5, Ventana] was used on the Ventana and Roche Benchmark Ultra automated slide stainer. The Her2 immunohistochemistry scoring was performed according to the American Society of Clinical Oncology and College of American Pathologists 2016 guidelines).

Osimertinib was discontinued, and the patient received carboplatin, etoposide, and atezolizumab. After three cycles, restaging revealed progressive pleural disease and multiple (>10) new brain metastases, prompting whole-brain radiotherapy. Subsequent systemic progression led to two cycles of cisplatin and paclitaxel, resulting in stable disease. Due to persistent *EGFR* exon 19 deletion in previous tumor tissue and cumulative chemotherapy toxicity, therapy was switched back to osimertinib.

**Figure 2:**
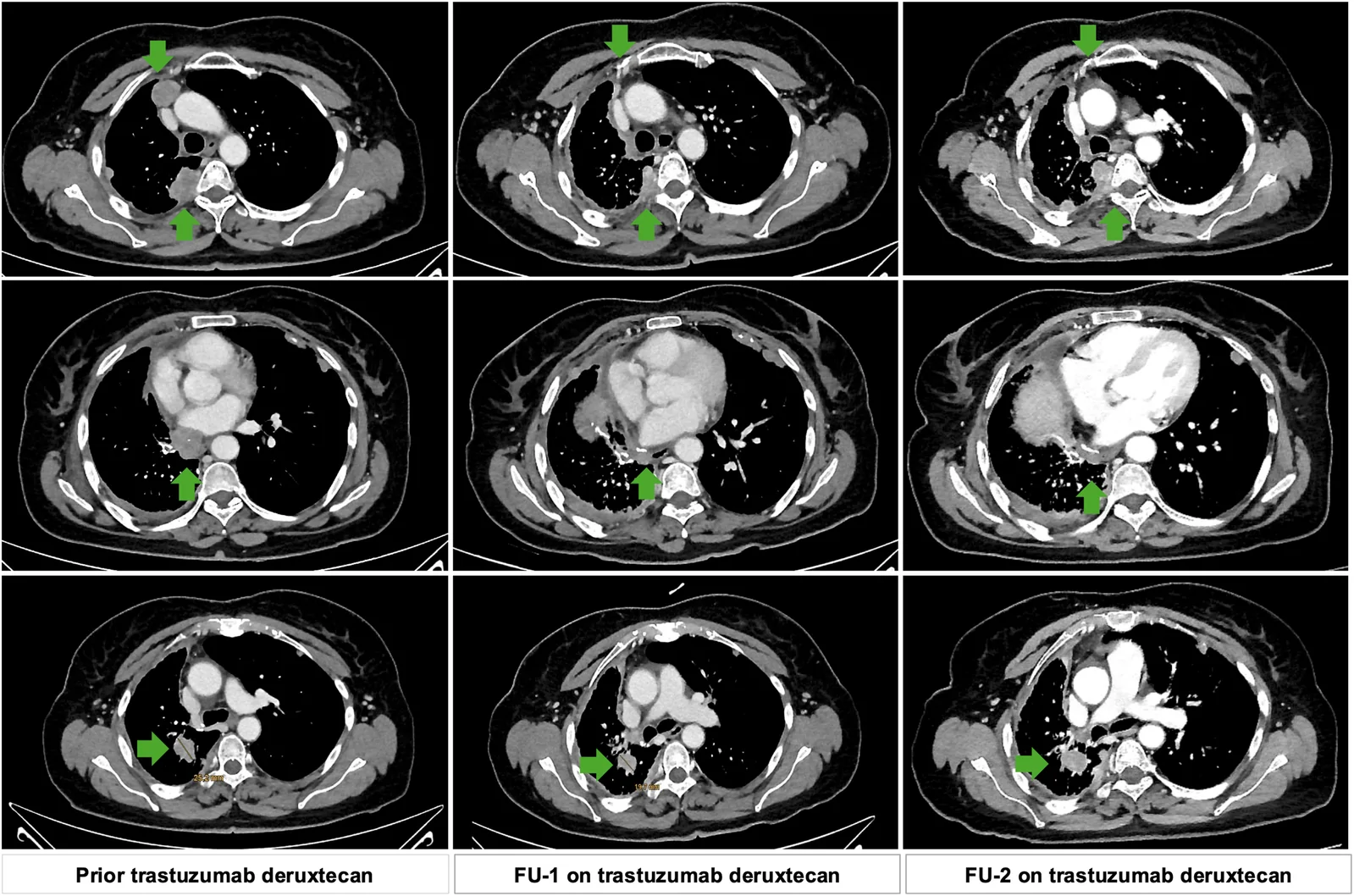
CT scan. Partial response to T-DXd according to RECIST v.1.1. The left column presents the CT scan before T-DXd initiation and the middle and right columns present the staging 8 and 16 weeks after initiation. Tumor shrinkage was observed in all target lesions: mediastinal lymph node (row 1), pleural metastasis (row 1), infrahilar lymph node (row 2), and lung metastasis in the right upper lobe (row 3). CT, computed tomography; FU, follow up; RECIST v.1.1, Response Evaluation Criteria in Solid Tumors version 1; T-DXd, trastuzumab deruxtecan.

In July 2025, progressive mediastinal lymphadenopathy, pulmonary nodules, and pleural carcinomatosis were noted, whereas brain metastases remained stable post–whole-brain radiotherapy. Based on the known *HER2* amplification, off-label therapy with the antibody-drug conjugate T-DXd (5.4 mg/kg every 3 wk) was initiated. After three cycles, CT imaging revealed partial response with regression of mediastinal, pulmonary, and pleural lesions (−40.1% per RECIST v.1.1; Fig. 2), and cranial magnetic resonance imaging result remained stable. Clinically, the patient experienced substantial relief from pleural carcinomatosis-associated pain.

After 90 days of treatment, new-onset dizziness and nausea prompted lumbar puncture, revealing leptomeningeal carcinomatosis with adenocarcinoma cells. After multidisciplinary risk-benefit assessment, particularly considering the high risk of treatment-related pneumonitis, osimertinib 160 mg once daily was combined with T-DXd. The associated pneumonitis risk was extensively discussed with the patient in a shared decision-making process. Neurologic symptoms initially improved, and imaging on November 5 revealed ongoing partial response (−31.1% per RECIST v.1.1; Fig. 2). By January 2026, meningeal symptoms worsened, and therapy was transitioned to best supportive care, with continuation of osimertinib 80 mg once daily to prevent tumor flare. The patient died on January 25, 2026.

## Discussion

SCT is a challenging resistance mechanism in *EGFR*-positive NSCLC, occurring in approximately 3% of patients treated with first- or second-generation TKIs and rising to 15% to 23% after third-generation TKI therapy.^1^ Prognosis after transformation is poor, with median progression-free survival of 2 to 5 months and overall survival of 9 to 13 months, irrespective of treatment with chemotherapy alone, chemoimmunotherapy, or chemotherapy combined with EGFR inhibitors.^1^^,^^2^ Consequently, evidence for optimal treatment is limited, with platinum and etoposide remaining the most frequently used backbone.

*HER2* amplification is a validated bypass mechanism of resistance to EGFR TKIs, occurring in approximately 2% to 7% of patients. Clinical experience suggests that patients with *EGFR*-resistant, *HER2*-amplified NSCLC may derive benefit from HER2-directed therapies, including irreversible pan-HER agents (e.g., afatinib) or combinations of EGFR TKIs and HER2 TKIs (e.g., gefitinib plus pyrotinib).^3^ Although T-DXd has demonstrated efficacy in *HER2*-mutant NSCLC and HER2-overexpressing tumors, its role in NSCLC with de novo or acquired *HER2* amplification remains to be elicited.^4^^,^^5^

The coexistence of SCT and *HER2* amplification in *EGFR*-resistant NSCLC has not been previously described. In our case, despite progression on multiple lines of therapy—including chemoimmunotherapy and platinum rechallenge—the patient achieved a deep but short clinical and radiographic response to T-DXd monotherapy, underscoring that molecularly actionable alterations may be therapeutically relevant despite lineage plasticity.

## Conclusion

This case illustrates *EGFR*-resistant NSCLC with SCT and co-occurring *HER2* amplification responding to T-DXd after failure on chemoimmunotherapy. It underscores the importance of repeat biopsy and comprehensive molecular profiling at progression to identify potential therapeutic opportunities in refractory disease.

## CRediT Authorship Contribution Statement

**Malte Verheyen:** Conceptualization, Data curation, Methodology, Resources, Visualization, Writing – original draft, Writing – review & editing.

**Lea Ruge:** Conceptualization, Data curation, Methodology, Resources, Visualization, Writing – original draft, Writing – review & editing.

**Anne Bunck:** Writing – review & editing, Visualization.

**Alexander Quaas:** Methodology, Writing – review & editing, Resources.

**Felix John:** Writing – review & editing, Resources.

**Lucia Nogova:** Writing – review & editing, Resources.

**Richard Riedel:** Writing – review & editing, Resources.

**Sofia Stilianakis:** Writing – review & editing, Resources.

**Heather Scharpenseel:** Writing – review & editing, Resources.

**Matthias Scheffler:** Writing – review & editing, Resources.

**Idil Ahmed:** Writing – review & editing, Resources.

**Carolin Jakob:** Writing – review & editing, Resources.

**Jan Philip Weber:** Writing – review & editing, Resources.

**Martin Sebastian:** Writing – review & editing, Resources.

**Fabian Acker:** Writing – review & editing, Resources.

**Jürgen Wolf:** Writing – review & editing, Resources.

**Sebastian Michels:** Writing – review & editing, Supervision.

## Disclosure

Dr. Acker received consulting fees from AstraZeneca, Daiichi Sankyo, IQVIA, Johnson & Johnson, Merck, Novartis, and NovoCure; payment or honoraria for lectures, presentations, or educational events from Amgen, AstraZeneca, BeOne, Johnson & Johnson, MedToday, NeoConnect, Regeneron, and RS GmbH; and support for attending meetings and/or travel from Amgen, AstraZeneca, and Daiichi Sankyo. Dr. Ahmed received consulting fees from Boehringer-Ingelheim and support for attending meetings and/or travel from Boehringer-Ingelheim. Dr. Jakob received support for attending meetings and/or travel from Stemline. Dr. John received grants or contracts from AstraZeneca and Amgen; consulting fees from Boehringer Ingelheim, Reesi, and Johnson & Johnson; and support for attending meetings and/or travel from Merck, Amgen, and Johnson & Johnson. Dr. Michels received grants or contracts from Else-Kröner Forschungskolleg Cologne, Dr. Rolf M. Schwiete Stiftung, Novartis, and Janssen-Cilag; payment or honoraria for lectures, presentations, or educational events from BeOne Medicines, Takeda, Bristol Myers Squibb, Janssen-Cilag, and Pfizer Oncology; and support for attending meetings and/or travel from BeOne Medicines, Janssen-Cilag, Takeda, and Eli Lilly and Company. Dr. Nogova received grants or contracts from Pfizer, Bristol Myers Squibb, Novartis, Merck Sharp & Dohme, Janssen, Amgen, Dracen, and Siemens; consulting fees from Roche, Janssen, Pfizer, and Bristol Myers Squibb; payment or honoraria for lectures, presentations, or educational events from Pfizer, Roche, Janssen, AstraZeneca, and Merck Sharp & Dohme; and support for attending meetings and/or travel from Janssen, Pfizer, and Roche. Dr. Riedel received consulting fees from Janssen and participated on advisory boards for Janssen. Dr. Ruge received consulting fees from Johnson & Johnson; payment or honoraria for lectures, presentations, or educational events from Amgen and Johnson & Johnson; and support for attending meetings and/or travel from Johnson & Johnson and Amgen. Dr. Scharpenseel received support for attending meetings and/or travel from Johnson & Johnson. Dr. Scheffler received consulting fees from Amgen, AstraZeneca, Bristol Myers Squibb, Boehringer Ingelheim, Daiichi Sankyo, Novartis, Pfizer, Roche, and Takeda; payment or honoraria for lectures, presentations, or educational events from Bristol Myers Squibb, Amgen, Boehringer Ingelheim, Daiichi Sankyo, Pfizer, Roche, Sanofi-Aventis, and Takeda; payment for expert testimony from Amgen, AstraZeneca, Janssen, Pfizer, and Takeda; support for attending meetings and/or travel from Johnson & Johnson and AstraZeneca; and other nonfinancial interests with Amgen (Coordinating PI, no financial interest), Boehringer Ingelheim (Steering Committee Member), Dracen (Local PI, no financial interest), Siemens Healthineers (Coordinating PI, no financial interest), ESMO Climate Change Task Force, Handelsblatt Health Circle, OncoEducation (PI; YouTube channel for patients), and Zielgenau (advisory role, patient advocacy). Dr. Sebastian received grants or contracts from AstraZeneca and Gilead; received support for attending meetings and/or travel from Pfizer, Takeda, Merck, and Daiichi Sankyo; participated on advisory boards for Amgen, Boehringer-Ingelheim, Pfizer, AstraZeneca, Gilead, Daiichi Sankyo, Sanofi, and Regeneron; and serves as board member of AIO and AIO-Studien gGmbH. Dr. Stilianakis received support for attending meetings and/or travel from Johnson & Johnson. Dr. Verheyen received payment or honoraria for lectures, presentations, or educational events from Pfizer, Johnson & Johnson, Bristol Myers Squibb, AbbVie, BeiGene, and Stemline and support for attending meetings and/or travel from BeiGene, Lilly, and Johnson & Johnson. Dr. Weber received consulting fees from Boehringer Ingelheim, AstraZeneca, Bristol Myers Squibb, Johnson & Johnson, Amgen, Accord Health, Novartis, Lilly, Ipsen, Hexal, MSD/Merck, Sobi, Reesi, and Daiichi Sankyo; payment or honoraria for lectures, presentations, or educational events from Boehringer Ingelheim, AstraZeneca, Bristol Myers Squibb, Johnson & Johnson, Amgen, Accord Health, Novartis, Lilly, Ipsen, Hexal, MSD/Merck, Sobi, Reesi, and Daiichi Sankyo; and support for attending meetings and/or travel from Boehringer Ingelheim, AstraZeneca, Bristol Myers Squibb, Johnson & Johnson, Amgen, Accord Health, Novartis, Lilly, Ipsen, Hexal, MSD/Merck, Sobi, Reesi, and Daiichi Sankyo. Dr. Wolf received grants or contracts from Amgen, AstraZeneca, Bristol Myers Squibb, Janssen Pharmaceutica, Novartis, and Pfizer; received payment or honoraria for lectures, presentations, or educational events from AbbVie, Amgen, AstraZeneca, BeiGene, Blueprint, Bristol Myers Squibb, Boehringer-Ingelheim, Chugai, Daiichi Sankyo, Disco Pharmaceuticals, Ellipses Pharma, Genmab, Janssen, Lilly, Loxo, Merck, Mirati, Merck Sharp & Dohme, Novartis, Nuvalent, Pfizer, Pierre-Fabre, Regeneron, Roche, Seattle Genetics, Takeda, Turning Point, and Zuelling Pharma; and participated on advisory boards for AbbVie, Amgen, AstraZeneca, BeiGene, Blueprint, Bristol Myers Squibb, Boehringer-Ingelheim, Chugai, Daiichi Sankyo, Disco Pharmaceuticals, Ellipses Pharma, Genmab, Janssen, Lilly, Loxo, Merck, Mirati, Merck Sharp & Dohme, Novartis, Nuvalent, Pfizer, Pierre-Fabre, Regeneron, Roche, Seattle Genetics, Takeda, Turning Point, and Zuelling Pharma. Dr. Quaas received grants or contracts from German Cancer Fund, German Research Fund, Wilhelm Sander Fund, Boll Foundation, Cologne Excellence Forum, and Cologne Fortune; payment or honoraria for lectures, presentations, or educational events from Bristol Myers Squibb, Merck Sharp & Dohme, Astellas, Amgen, Jazz, onkowissen, Servier, Incyte, Beigene, and AstraZeneca; and support for attending meetings and/or travel from Bristol Myers Squibb, Merck Sharp & Dohme, Astellas, Amgen, Jazz, Servier, Incyte, Beigene, and AstraZeneca. The remaining authors declare no conflict of interest.
